# Efficient and Selective Extraction of Rhamnogalacturonan-I-Enriched Pectic Polysaccharides from Tartary Buckwheat Leaves Using Deep-Eutectic-Solvent-Based Techniques

**DOI:** 10.3390/foods13040625

**Published:** 2024-02-19

**Authors:** Ding-Tao Wu, Jing Lei, Jie Li, Mei-Mei Qu Mo, Wen-Bing Li, Yu-Jing Huang, Yi-Chen Hu, Ai-Li Wang, Liang Zou

**Affiliations:** 1 Key Laboratory of Coarse Cereal Processing (Ministry of Agriculture and Rural Affairs), Sichuan Engineering & Technology Research Center of Coarse Cereal Industralization, School of Food and Biological Engineering, Chengdu University, Chengdu 610106, China; 2 Tibetan Plateau Ethnic Medicinal Resources Protection and Utilization Key Laboratory of National Ethnic Affairs Commission of the People’s Republic of China, Sichuan Provincial Qiang-Yi Medicinal Resources Protection and Utilization Technology Engineering Laboratory, Southwest Minzu University, Chengdu 610225, China

**Keywords:** Tartary buckwheat, deep eutectic solvent, high-pressure processing, rhamnogalacturonan-I-enriched pectic polysaccharide, biological activity

## Abstract

Tartary buckwheat green leaves are considered to be among the most important by-products in the buckwheat industry. Although Tartary buckwheat green leaves are abundant in pectic polysaccharides, their potential applications in the food industry are quite scarce. Therefore, to promote their potential applications as functional or fortified food ingredients, both deep-eutectic-solvent-assisted extraction (DESE) and high-pressure-assisted deep eutectic solvent extraction (HPDEE) were used to efficiently and selectively extract pectic polysaccharides from Tartary buckwheat green leaves (TBP). The results revealed that both the DESE and HPDEE techniques not only improved the extraction efficiency of TBP but also regulated its structural properties and beneficial effects. The primary chemical structures of TBP extracted using different methods were stable overall, mainly consisting of homogalacturonan and rhamnogalacturonan-I (RG-I) pectic regions. However, both the DESE and HPDEE methods could selectively extract RG-I-enriched TBP, and the proportion of the RG-I pectic region in TBP obviously improved. Additionally, both the DESE and HPDEE methods could improve the antioxidant and anti-glycosylation effects of TBP by increasing its proportion of free uronic acids and content of bound polyphenolics and reducing its molecular weight. Moreover, both the DESE and HPDEE methods could partially intensify the immunostimulatory effect of TBP by increasing its proportion of the RG-I pectic region. These findings suggest that DES-based extraction techniques, especially the HPDEE method, can be promising techniques for the efficient and selective extraction of RG-I-enriched TBP.

## 1. Introduction

Pectic polysaccharides are abundant inside plant cell walls, which consist of various structurally distinct regions or domains, mainly homogalacturonan (HG), rhamnogalacturonan-I (RG-I), and rhamnogalacturonan-II (RG-II) [1,2,3]. The HG region usually consists of a linear backbone of →4)-α-D-GalA*p*-(1→ residues that are partially methyl-esterified and O-acetylated, accounting for about 65% of pectic polysaccharides. In addition, the RG-I region is usually composed of a backbone of →4)-α-D-GalA*p*-(1→2,4)-α-L-Rha*p*-(1→ disaccharide residues with neutral side chains, accounting for about 20–35% of pectic polysaccharides [4]. Generally, commercial pectic polysaccharide mainly consists of an HG region with a small amount of RG-I domain, which is widely utilized as a thickener and gel in the food, medicine, and cosmetics industries [5,6]. However, recent experimental results have demonstrated that RG-I-enriched pectic polysaccharides exhibit several unique functions superior to HG-enriched pectic polysaccharides, such as anti-cancer, immunomodulatory, and prebiotic properties [2,6]. Therefore, the preparation of RG-I-enriched pectic polysaccharides from edible plants and their favorable applications in the functional food industry are becoming increasingly attractive.

Tartary buckwheat (*Fagopyrum tataricum* Gaertn) is a gluten-free pseudocereal of the genus *Fagopyrum*, which is widely distributed in China and Russia [7,8]. Its seeds constitute a highly nutritional cereal, which has a plethora of functional components (e.g., dietary polysaccharides, polyphenols, and proteins) [7,8]. As a consequence, its seeds have various health-promoting effects owing to their abundant functional ingredients [8]. In China, Tartary buckwheat seeds are widely consumed as raw materials of functional tea beverages [8]. In fact, like Tartary buckwheat seeds, Tartary buckwheat green leaves, some of the most important by-products in the buckwheat industry, also have abundant functional components, and these leaves are also utilized as raw materials in functional tea beverages. Interestingly, compared to the seeds, the green leaves are much richer in pectic polysaccharides, primarily consisting of HG and RG-I domains. Moreover, their main constituent monosaccharides are galacturonic acid (GalA), galactose (Gal), arabinose (Ara), and rhamnose (Rha), and their molecular masses are in the range of 5.803 × 10^5^–6.975 × 10^5^ Da [9]. Furthermore, pectic polysaccharides from Tartary buckwheat green leaves also exhibit health-promoting benefits, e.g., in vitro antioxidant, antiglycation, anticancer, prebiotic, and anti-hyperlipidemia effects [9]. Nevertheless, studies on pectic polysaccharides from Tartary buckwheat green leaves and their potential applications as functional or fortified food ingredients are still scant. So, to promote their potential application, it is necessary to develop green and efficient methods for preparing pectic polysaccharides from Tartary buckwheat green leaves.

Generally, the conventional hot water extraction (CHWE) method is considered the common approach for industrialized pectic polysaccharide preparation; nevertheless, it always requires a long extraction time to attain a good extraction yield [4]. Although alkaline and acidic buffers are usually added into the water to reduce processing time and regulate the physicochemical properties of pectic polysaccharides, these buffers are always environmentally unfriendly [3,4]. Deep eutectic solvents (DESs) are attracting increasing interest in relation to their application as an alternative extraction solvent for efficiently preparing pectic polysaccharides from edible plants [10,11,12,13]. A DES is a new green extraction solvent that is composed of a hydrogen bond acceptor and a hydrogen bond donor, providing additional interactions with plant cell walls and subsequently allowing more pectic polysaccharides to be extracted compared to conventional extraction solvents [10,13,14,15]. Furthermore, DESs also offer several advantages, e.g., inexpensiveness, biodegradability, and eco-friendliness [11,16], and pectic polysaccharides extracted by DESs usually provide superior health benefits [13,15,17,18]. Furthermore, apart from the extraction solvent used, several innovative methods, e.g., ultrasound extraction, microwave extraction, and high-pressure processing extraction, are also applied to improve the extraction rates and health benefits of pectic polysaccharides [4]. In particular, as an important non-thermal, eco-friendly, and green processing technique, high-pressure processing extraction can enhance the extraction rate for targeting RG-I-enriched pectic polysaccharides from fruits and their by-products [19,20,21]. Therefore, we speculated that the combined utilization of a DES and high pressure could be a promising approach for the efficient and selective extraction of RG-I-enriched pectic polysaccharides from edible plants.

This study aimed to optimize the extraction conditions of both deep-eutectic-solvent-assisted extraction (DESE) and high-pressure-assisted deep eutectic solvent extraction (HPDEE) for the efficient preparation of pectic polysaccharides from Tartary buckwheat green leaves (TBP) and to further reveal the corresponding influences on the structural properties and biological effects of TBP. The findings can provide innovative approaches for the selective extraction of rhamnogalacturonan-I-enriched TBP and are beneficial to enhancing their potential applications as functional or fortified food ingredients.

## 2. Materials and Methods

### 2.1. Materials and Chemicals

Tartary buckwheat green leaves (*F. tataricum* cv. Chengku No. 2) were collected from buckwheat plants after two months of seeding in the buckwheat breeding and cultivation base of Chengdu University (GPS coordinate 104°56′59″ N, 30°32′90″ E). Tartary buckwheat green leaves were vacuum freeze-dried at −80 °C for 48 h and then ground into a powder and sieved through a 50-mesh screen.

2,2′-azino-bis-(3-ethylbenzothiazoline-6-sulfonic acid) (ABTS), 2,2-diphenyl-1-picrylhydrazyl (DPPH), and 1-pheny-3-methyl-5-pyrazolone (PMP) were acquired from Aladdin-E (Shanghai, China). Monosaccharide standards, namely, rhamnose (Rha), mannose (Man), glucuronic acid (GlcA), galacturonic acid (GalA), glucose (Glc), galactose (Gal), xylose (Xyl), and arabinose (Ara), were acquired from Sigma-Aldrich (St. Louis, MO, USA). ELISA kits for the determination of tumor necrosis factor-alpha (TNF-α) and interleukin-6 (IL-6) levels and characteristics were acquired from Elabscience (Wuhan, China).

### 2.2. Extraction and Isolation of Pectic Polysaccharides from Tartary Buckwheat Green Leaves

#### 2.2.1. Pre-Treatment of Raw Materials

In order to remove ethanol-soluble substances in the Tartary buckwheat green leaves before the extraction of pectic polysaccharides, the powder of dried Tartary buckwheat green leaves was mixed with ethanol at a concentration of 80% (*v*/*v*) in a ratio of 1:10 (g/mL). Subsequently, the mixtures were extracted using an ultrasonic cleaning machine (SB-800DTD, Ningbo Scientz Biotechnology Co., Ltd., Ningbo, China) operated at 480 W for 30 min to eliminate the majority of ethanol-soluble constituents. Afterward, the extracted residues were dried in a drying oven at 45 °C (Shanghai Boxun Industry and Commerce Co., Ltd., Shanghai, China) and subsequently utilized for the extraction of pectic polysaccharides in the following steps.

#### 2.2.2. Conventional Hot-Water Extraction (CHWE)

CHWE was performed based on a previously documented method, with minor modifications [9]. Accordingly, the extracted residues of Tartary buckwheat green leaves (5.0 g) were treated with deionized water (1:30, g/mL) at 95 °C for 180 min. After centrifugation (4000× *g*, 15 min, 4 °C), the supernatant was obtained and concentrated, and the starch was removed via enzymatic degradation with both α-amylase (5 U/mL and 6 h) and glucoamylase (5 U/mL and 8 h). After the removal of starch, the supernatant (about 100 mL) was applied for the precipitation of pectic polysaccharides by adding four volumes (about 400 mL) of 95% (*v*/*v*) ethanol. Furthermore, the precipitations were dissolved in ultrapure water. To completely eliminate small molecules (e.g., monosaccharides, oligosaccharides, and hydrolysates derived from starch), ultrafiltration (molecular weight cutoff, 3500 Da) was carried out, and the supernatant was ultra-filtered three times. Finally, pectic polysaccharides from Tartary buckwheat green leaves (TBP) extracted via CHWE were pre-frozen at −80 °C ± 2 °C for 8 h, dried using vacuum freeze-drying at −80 °C ± 2 °C for 48 h, and named TBP-W.

#### 2.2.3. Deep-Eutectic-Solvent-Assisted Extraction (DESE)

A deep eutectic solvent (DES) storage solution was synthesized by mixing choline chloride and ethylene glycol (molar ratio, 1:3) based on a previously documented method [16]. Furthermore, the DES storage solution was diluted by adding certain volumes of deionized water to prepare a DES extraction solvent. The extracted residues of Tartary buckwheat green leaves (about 5.0 g) were mixed with different volumes of DES extraction solution and then extracted at different temperatures for specific extraction times. Afterward, the next steps in the preparation of pectic polysaccharides from Tartary buckwheat green leaves (TBP) were conducted, constituting the same steps as discussed in Section 2.2.2, and TBP extracted via DESE was named TBP-D. For the optimization of DESE, single-factor experimental design (SFD) was applied to assess the impact of the water content in the DES solvent (10–70%), extraction time (90–210 min), liquid-to-solid ratio (20–60 mL/g), and extraction temperature (60–100 °C) on the extraction yield of TBP-D. Moreover, a Box–Behnken design (BBD) was also employed to assess the impacts of independent factors on the yields of TBP-D. The experimental runs and corresponding results are shown in Table 1. Finally, the experimental results were investigated and modeled using a second-order polynomial equation.

#### 2.2.4. High-Pressure-Assisted Deep Eutectic Solvent Extraction (HPDEE)

The HPDEE was carried out using an experimental-level ATS high-pressure homogenizer (AH-NANO, ATS Engineering Limited (Suzhou), Suzhou, China) at room temperature. The DES extraction solvent was prepared in accordance with Section 2.2.3. The extracted residues of Tartary buckwheat green leaves (about 5.0 g) were mixed with different volumes of DES extraction solution and then extracted under different pressures for certain extraction time. Afterward, the next steps for the preparation of pectic polysaccharides from Tartary buckwheat green leaves (TBP), the same steps as those discussed in Section 2.2.2, were carried out, and the TBP extracted by HPDEE was named TBP-PD. For the optimization of HPDEE, the SFD was also used to assess the impacts of extraction time (7–19 min), water content in DES solvent (10–70%), liquid-to-solid ratio (20–60 mL/g), and extraction pressure (50–130 MPa) on the extraction yield of TBP-PD. Similarly, a BBD design was also employed to evaluate the impacts of extraction factors on the yields of TBP-PD. The experimental runs and corresponding results are shown in Table 1. The experimental results were investigated and modeled by using a second-order polynomial equation.

### 2.3. Physicochemical and Structural Characterization of Pectic Polysaccharides from Tartary Buckwheat Green Leaves Extracted Using Different Techniques

The quantification of total polysaccharides, acidic sugars, proteins, and bound polyphenolics in TBP-W, TBP-D, and TBP-PD was conducted using colorimetric methods performed as described in previous studies [9,22]. The molecular masses (*M_w_*) and molecular mass distributions (*M_w_*/*M_n_*) of TBP-W, TBP-D, and TBP-PD were measured via size exclusion chromatography combined with multiangle laser light scattering detection and refractive index detection (Wyatt Technology Co., Santa Barbara, CA, USA), as previously documented [22]. The primary chemical structures of TBP-W, TBP-D, and TBP-PD were elucidated using Fourier transform infrared spectroscopy (FT-IR) analysis and nuclear magnetic resonance spectroscopy (NMR) analysis, as previously reported [9,22]. Briefly, the monosaccharide units of TBP-W, TBP-D, and TBP-PD were measured via PMP-derivatization followed by liquid chromatography analysis (LC, L-20A, Shimadzu, Japan). The chemical groups and esterified degrees of TBP-W, TBP-D, and TBP-PD were analyzed using FT-IR analysis (Spectrum Two, PerkinElmer, Waltham, MA, USA). The glycosidic linkages of TBP-W, TBP-D, and TBP-PD were analyzed utilizing a Bruker Ascend 600 MHz spectrometer (Bruker, Rheinstetten, Germany).

### 2.4. Evaluation of Beneficial Effects of Pectic Polysaccharides from Tartary Buckwheat Green Leaves Extracted Using Different Techniques

The free-radical-scavenging abilities (ABTS and DPPH) and ferric-reducing antioxidant powers (FRAP) of TBP-W, TBP-D, and TBP-PD were determined to assess their antioxidant effects [9,22]. The scavenging abilities of TBP extracted via different techniques were assessed at various concentrations, and the IC_50_ values (mg/mL) of TBP-W, TBP-D, and TBP-PD were calculated separately. In addition, the inhibitory effects of TBP-W, TBP-D, and TBP-PD against the generation of advanced glycation end-products (AGEs) in an in vitro bovine serum albumin–glucose model were determined to assess their anti-glycosylation effects [9]. Furthermore, the immunostimulatory properties of TBP-W, TBP-D, and TBP-PD were assessed by using an in vitro cell model [22]. Effects of TBP-W, TBP-D, and TBP-PD on the release of nitric oxide (NO), IL-6, and TNF-α from RAW 264.7 macrophages were assessed.

### 2.5. Statistical Analysis

All data were analyzed using Design Expert software (v8.0.6.1, Stat-Ease Inc., Minneapolis, MN, USA) and IBM SPSS Statistics software (v22.0, SPSS, Inc., Chicago, IL, USA). One-way analysis of variance and Student’s *t*-test were carried out to assess statistical significance (*p* < 0.05).

## 3. Results and Discussion

### 3.1. Extraction Optimization of Deep-Eutectic-Solvent-Assisted Extraction (DESE) and High-Pressure-Assisted Deep Eutectic Solvent Extraction (HPDEE)

#### 3.1.1. Extraction Optimization for the DESE Method

**Figure 1:**
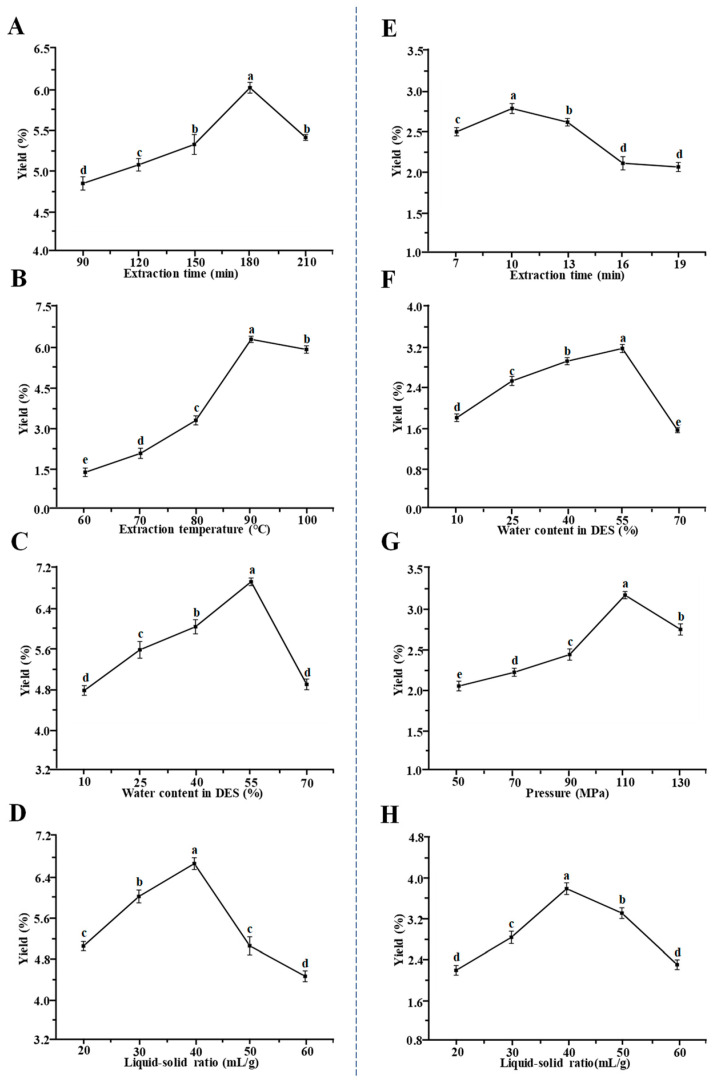
Impacts of various extraction factors of deep-eutectic-solvent-assisted extraction (**A**–**D**) and high-pressure-assisted deep eutectic solvent extraction (**E**–**H**) methods on the yields of pectic polysaccharides from Tartary buckwheat green leaves. (**A**–**D**) indicate the effects of extraction time, extraction temperature, water content in DES, and liquid-solid ratio on the yields of pectic polysaccharides, respectively. (**E**–**H**) indicate the effects of extraction time, water content in DES, extraction pressure, and liquid-solid ratio on the yields of pectic polysaccharides, respectively. The error bars are standard deviations. Significant differences (*p* < 0.05) among the yields obtained under different extraction conditions are indicated by data bearing different letters (a–e).

Figure 1 illustrates the impacts of various extraction factors, namely, time, temperature, the content of water in the deep eutectic solvent (DES), and the liquid-to-solid ratio, on the extraction yields of TBP-D. Specifically, the yields of TBP-D exhibited an upward trend as the extraction time increased from 90 to 180 min, but the yields declined past this point as time went on (Figure 1A). This result might be due to the thermal degradation of pectic polysaccharides during the long heating process [4]. In addition, temperature was also an important factor influencing the yield of pectic polysaccharides [5]. The extraction yield reached a maximum value when the temperature was 90 °C (Figure 1B). It is important to note that a higher temperature might also result in the thermal degradation of pectic polysaccharides. Furthermore, the water content in a DES significantly affects the physical properties of a DES solution, resulting in various extraction yields of pectic polysaccharides [13]. As presented in Figure 1C, the extraction yields of TBP-D exhibited a positive correlation with the content of water ranging from 10% to 55%, while the yields of TBP-D declined with the increased water content. Generally, the low water content in a DES can make it difficult to penetrate into plant cell walls due to its high viscosity, while high water content can block the interactions between pectic polysaccharides and the DES [13,23]. Moreover, as represented in Figure 1D, the yield of TBP-D reached a maximum value when the liquid-to-solid ratio was 40 mL/g. Finally, the optimal time, temperature, water content in the DES, and liquid-to-solid ratio were determined to be 180 min, 90 °C, 55%, and 40 mL/g, respectively.

Table 1 displays the BBD matrix and outcomes, and the predictive mathematical model is a second-order polynomial equation:

(1) $$\begin{array}{c} Y_{1}=7.39+0.2608X_{11}-0.06000X_{12}-0.3825X_{13}+1.21X_{14}+0.2725X_{11}X_{12}\\ \;\;\;\;\;\;\;\;\;\;\;\;\;\;\;\;\;\;\;\;\;\;\;\;\;\;\;\;+0.2200X_{11}X_{13}-0.0800X_{11}X_{14}-0.2475X_{12}X_{13}\\ \;\;\;\;\;\;\;\;\;\;\;\;\;\;\;\;\;\;\;\;\;\;\;\;\;\;\;\;+0.1550X_{12}X_{14}+0.0400X_{13}X_{14}-0.6142X_{11}^{2}-1.49X_{12}^{2}\\ \;\;\;\;\;\;\;\;\;\;\;\;\;\;\;\;\;\;\;\;\;\;\;\;\;\;\;\;-1.00X_{13}^{2}-1.56X_{14}^{2} \end{array}$$

where Y_1_ is the yield of TBP-D, while X_11_, X_12_, X_13_, and X_14_ are the time (min), water content in the DES (%), the liquid-to-solid ratio (mL/g), and temperature (°C), respectively.

**Figure 2:**
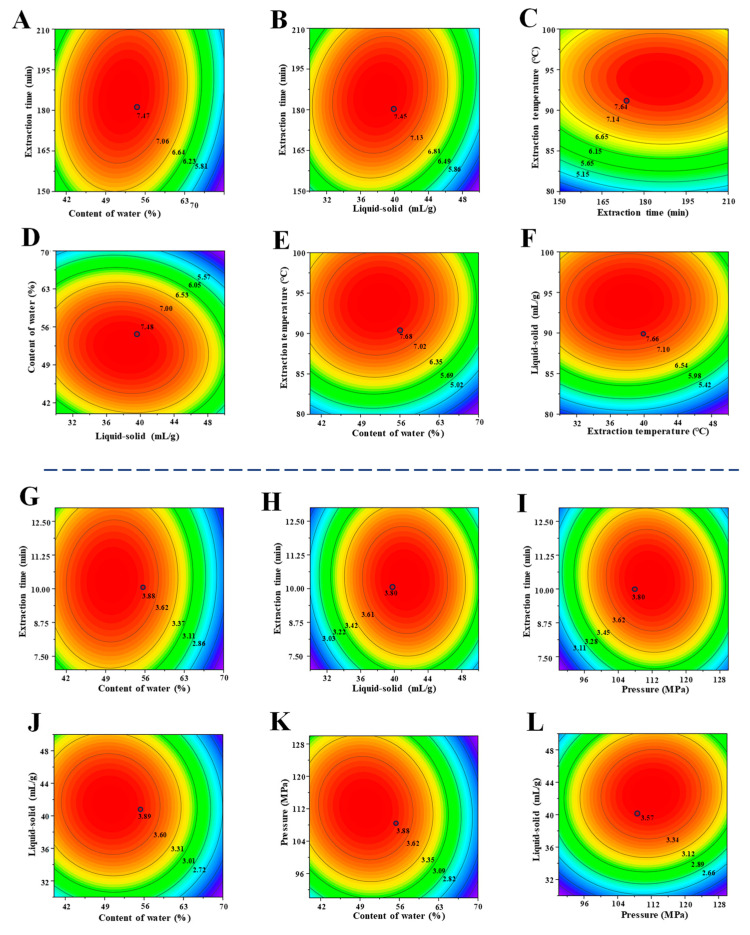
Two-dimensional contour plots of deep-eutectic-solvent-assisted extraction (**A**–**F**) and high-pressure-assisted deep eutectic solvent extraction (**G**–**L**). (**A**–**F**) indicate interactions among extraction time, water content in DES, extraction temperature, and liquid/solid ratio, respectively. (**G**–**L**) indicate interactions among extraction time, water content in DES, extraction pressure, and liquid–solid ratio, respectively.

According to the outcomes presented in Table 2, the predictive model for the DESE method was significant and could adequately predict the yields of TBP-D [23,24]. In addition, the predictive model had good repeatability, good reliability, and sufficient precision according to the values of the coefficient of variation, adequate precision, R^2^, and adjusted R^2^ (Table 2) [25,26]. Furthermore, two-dimensional (2D) contour plots of the predictive model are shown in Figure 2. As shown in Figure 2, the interaction effects between the extraction time and water content in the DES, extraction time and the liquid-to-solid ratio, and the water content in the DES and liquid-to-solid ratio were significant, constituting the same effects as those shown in Table 2. By analyzing the equations, the following predictive conditions were obtained: a time of 189.71 min, a water content in the DES of 51.73% (*v*/*v*), a liquid-to-solid ratio of 36.68 mL/g, and a temperature of 92.61 °C. In fact, the validation experiments were conducted using a time of 190.0 min, a water content in the DES of 52.0% (*v*/*v*), a liquid-to-solid ratio of 37.0 mL/g, and a temperature of 93.0 °C, and the observed yield of TBP-D was 7.59% ± 0.09% (n = 3), demonstrating a satisfactory level of concordance with the predictive yield (7.61%). Furthermore, the yield of TBP-D extracted via DESE was extremely higher than that of TBP-W (3.21% ± 0.06%) extracted via CHWE (Table 3), which might be attributed to the fact that the DES could provide additional interactions with the plant cell walls and, subsequently, that more pectic polysaccharides could be extracted [10,13]. These findings indicate that the DESE method can be used as an effective approach for the preparation of TBP.

#### 3.1.2. Extraction Optimization for the HPDEE Method

Figure 1 also illustrates the impacts of various extraction factors, namely, time, water content in the DES, liquid-to-solid ratio, and extraction pressure, on the yields of TBP-PD. The results revealed that the yields of TBP-PD extracted using HPDEE under different extraction times, water proportions in the DES, and liquid-to-solid ratios exhibited similar change trends to those of TBP-D extracted via DESE. In addition, the effect of high pressure on the yields were determined, as presented in Figure 1G. The yields of TBP-PD enhanced when the pressure increased from 50 MPa to 110 MPa, while obviously declining with a further increase in pressure. This result could be due to the physical degradation of pectic polysaccharides under excessively high pressure [27]. Finally, the optimal time, water content in the DES, pressure, and solid/liquid ratio were determined to be 10 min, 55%, 110 MPa, and 40 mL/g, respectively.

In addition, based on the experimental outcomes (Table 1), the predictive mathematical model was also a second-order polynomial equation, as shown below:

(2) $$\begin{array}{c} Y_{2}=3.91+0.0992X_{21}-0.5417X_{22}+0.1867X_{23}+0.1542X_{24}+0.0475X_{21}X_{22}-0.0525X_{21}X_{23}\\ \;\;\;\;\;\;\;\;\;\;\;\;\;\;\;\;\;\;\;\;\;\;\;\;\;\;-0.0775X_{21}X_{24}-0.0675X_{22}X_{23}-0.1150X_{22}X_{24}+0.0850X_{23}X_{24}-0.04344X_{21}^{2}\\ \;\;\;\;\;\;\;\;\;\;\;\;\;\;\;\;\;\;\;\;\;\;\;\;\;\;-0.8282X_{22}^{2}-0.7407X_{23}^{2}-0.6344X_{24}^{2} \end{array}$$

where Y_2_ is the yield of TBP-PD, and X_21_, X_22_, X_23_, and X_24_ are the time (min), water content in the DES solvent (%), liquid-to-solid ratio (mL/g), and pressure (MPa), respectively.

Furthermore, the predictive model for the HPDEE method was also highly statistically significant (*p* < 0.0001) according to the outcomes presented in Table 2. Furthermore, the predictive equation for the HPDEE method also had good repeatability, good reliability, and sufficient precision, which allowed it to adequately predict the yields of TBP-PD. Furthermore, according to the combination of the ANOVA analysis results (Table 2) and the 2D contour plots (Figure 2) of the predictive model, the interactions among different extraction parameters (X_21_X_22_, X_21_X_23_, X_22_X_23_, X_21_X_24_, X_22_X_24_, and X_23_X_24_) were significant (*p* < 0.05). All the data showed that the time, water content in the DES, liquid-to-solid ratio, and pressure were decisive factors for the TBP-PD extraction. By analyzing the experimental outcomes, the predictive conditions were determined, namely, a time of 9.46 min, a water content in the DES of 49.20% (*v*/*v*), a liquid-to-solid ratio of 40.99 mL/g, and a pressure of 117.05 MPa. Indeed, under the optimal conditions (a time of 10.0 min, a water content in the DES of 50.0% (*v*/*v*), a liquid-to-solid ratio of 41.0 mL/g, and a pressure of 110.0 MPa), the observed yield of TBP-PD was 3.94% ± 0.10% (n = 3), exhibiting a favorable level of concordance with the predicted yield of 3.98%. In addition, the yield of TBP-PD was also higher than that of TBP-W (3.21% ± 0.06%) in this study (Table 3). Meanwhile, the extraction time for the HPDEE method was only 10.0 min, which was significantly shorter than that of the CHWE (180 min), indicating that the optimized HPDEE method exhibited superior efficiency in comparison to the CHWE method. Furthermore, although the extraction yield of TBP-PD was lower than that of TBP-D, the time required for the HPDEE method (10 min) was also much shorter than that of the DESE method (190 min).

### 3.2. Physicochemical and Structural Properties of Pectic Polysaccharides from Tartary Buckwheat Green Leaves Extracted Using Different Methods

#### 3.2.1. Comparison of Chemical Components of TBP-W, TBP-D, and TBP-PD

Generally, a favorable extraction technology can not only improve the yield but also regulate the physicochemical properties and improve the functional properties of natural polysaccharides. Therefore, to reveal the influences of various extraction methods on the physicochemical and structural properties of TBP, the chemical components of TBP-W, TBP-D, and TBP-PD were detected and analyzed. The findings showed that various extraction methods not only notably affected the extraction yield of TBP but also obviously influenced its chemical components (Table 3). More specifically, it was revealed that the levels of polysaccharides in TBP-W, TBP-D, and TBP-PD ranged from 88.75 mg/100 mg to 94.48 mg/100 mg, and both TBP-D and TBP-PD had higher total quantities of polysaccharides than TBP-W. Furthermore, the total uronic acids in TBP-W, TBP-D, and TBP-PD ranged from 38.68 mg/100 mg to 47.67 mg/100 mg, and the lowest content was found in TBP-PD. A previous study revealed that TBP is mainly composed of HG and RG-I domains [9]. Therefore, compared to TBP-W, the lower uronic acid levels observed in TBP-PD might be attributed to the increased proportion of the RG-I pectic domain, suggesting that the HPDEE method might selectively extract RG-I-enriched pectic polysaccharides. In fact, previous studies have demonstrated that the high-pressure-assisted extraction method can accelerate the release of RG-I-enriched pectic polysaccharides from edible plants [19,20]. TBP-PD was identified as constituting RG-I-enriched pectic polysaccharides in later studies (Section 3.2.2). In addition, minor proteins were found in TBP-W, TBP-D, and TBP-PD, with the levels ranging from 1.49 mg/100 mg to 1.95 mg/100 mg, indicating that the influence of proteins on the beneficial effect of TBP could be neglected. Moreover, although polyphenolics were removed through the combined use of ultrasound-assisted ethanol extraction, ethanol precipitation, and membrane fractionation, few bound polyphenolics were still found in TBP-W, TBP-D, and TBP-PD, with the levels ranging from 4.36 mg GAE/g to 23.63 mg GAE/g. Compared to TBP-W, the higher number of bound polyphenolics observed in TBP-D might be due to the favorable solubility of polyphenolics in the DES [28]. In fact, the bound polyphenolics had positive effects on the biological functions of pectic polysaccharides [29,30].

#### 3.2.2. Comparison of Molecular Masses and Monosaccharide Compositions of TBP-W, TBP-D, and TBP-PD

**Figure 3:**
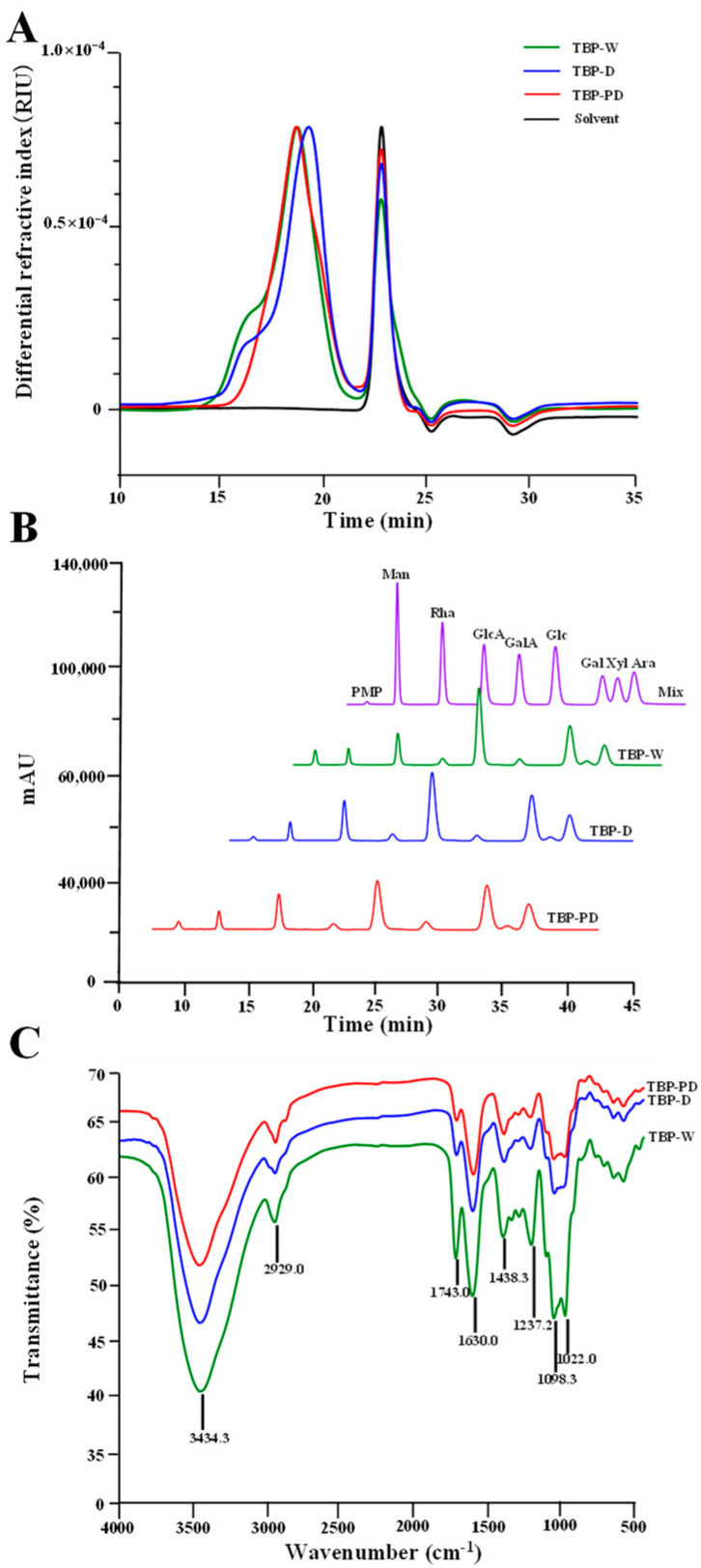
Size exclusion chromatography profiles (**A**), monosaccharide compositions (**B**), and Fourier transform infrared spectra (**C**) of TBP-W, TBP-D, and TBP-PD. TBP-W, TBP-D, and TBP-PD indicate pectic polysaccharides extracted from Tartary buckwheat green leaves via conventional hot water extraction, deep-eutectic-solvent-assisted extraction, and high-pressure-assisted deep eutectic solvent extraction, respectively. Man, mannose. Rha, rhamnose. GlcA, glucuronic acid. GalA, galacturonic acid. Glc, glucose. Gal, galactose. Xyl, xylose. Ara, arabinose.

To further understand the impacts of various extraction techniques on the structural properties of TBP, the molecular weights, monosaccharide units, chemical groups, and glycosidic bonds in TBP-W, TBP-D, and TBP-PD were detected and compared. The size exclusion chromatograms of TBP-W, TBP-D, and TBP-PD are shown in detail in Figure 3A. It can be seen that TBP-W, TBP-D, and TBP-PD exhibited distinct chromatograms. Compared to TBP-W, the chromatogram of TBP-D shows an obvious shift to the right, suggesting that the molecular weight of TBP-D extracted via DESE was lower than that of TBP-W. Notably, only TBP-PD possessed a symmetrical polysaccharide fraction, suggesting that the HPDEE method could selectively extract distinctive polysaccharide fraction with a reduced content of uronic acids from Tartary buckwheat green leaves (Table 3). In fact, TBP-PD was identified as consisting of RG-I-enriched pectic polysaccharides in the later studies. In addition, the molecular weights of TBP-W, TBP-D, and TBP-PD were determined to be 1.29 × 10^5^ Da, 0.67 × 10^5^ Da, and 0.88 × 10^5^ Da, respectively. These findings show that the molecular mass of TBP-W was notably higher than the molecular masses of TBP-D and TBP-PD, similar to previous reports showing that polysaccharides extracted using DES-based methods had lower molecular weights than that of CHWE [13,15,21]. Furthermore, the polydispersity (*M_w_*/*M_n_*) of TBP-PD was lower than that of TBP-W and TBP-D, suggesting that TBP-PD exhibited a relatively uniform molecular weight distribution.

A monosaccharide composition analysis was carried out to enable the structural characterization of TBP prepared using various methods. As displayed in Figure 3B, the monosaccharide units were identical for TBP-W, TBP-D, and TBP-PD, mainly consisting of GalA, Gal, Ara, and Rha, which confirmed that all the TBP types were pectic polysaccharides rich in homogalacturonan (HG) and rhamnogalacturonan-I (RG-I) domains. Nevertheless, the monosaccharide ratio was distinct for TBP extracted using different techniques. As presented in Table 3, the molar ratios of GalA, Gal, Ara, Rha, Xyl, Glc, GlcA, and Man in TBP-W, TBP-D, and TBP-PD were determined to be 3.35:1.83:1.00:1.00:0.14:0.22:0.32:0.32, 2.34:1.61:0.98:1.00:0.16:0.19:0.25:0.31, and 1.71:1.56:0.97:1.00:0.17:0.24:0.22:0.31, respectively. It is well known that the main monosaccharide unit in the HG domain is GalA, and the main monosaccharide units in the RG-I domain are GalA, Rha, Gal, and Ara [4,19]. Furthermore, the molar ratio of Rha/GalA (MR1) can reflect the proportion of RG-I and HG domains in pectic polysaccharides [3,4]. Notably, compared to TBP-W (GalA, 3.35; Rha/GalA, 0.30), the molar ratio of GalA in both TBP-D and TBP-PD decreased to 2.34 and 1.71, respectively, and their molar ratios of Rha/GalA increased to 0.43 and 0.58, respectively (Table 3). These findings showed that both the DESE and HPDEE methods could successfully applied to obtain RG-I-enriched TBP. The improvement in the RG-I domain in TBP-D could be due to the degradation of the HG backbone due to a β-elimination reaction [31]. Furthermore, the HG domain in pectic polysaccharides can also be destroyed under high-pressure treatment, resulting in an increase in the RG-I pectic domain [19,20,21]. Furthermore, the molar ratio of (Ara + Gal)/Rha (MR2) can reflect the extent of the branching degree of the RG-I pectic domain, and a high value indicates that the RG-I domain has many and/or long side chains [19]. Compared to TBP-W (2.83), the molar ratios of (Ara + Gal)/Rha in both TBP-D and TBP-PD slightly decreased to 2.59 and 2.53, indicating that both the DESE and HPDEE methods cause slight degradation of neutral side chains. Collectively, these findings suggest that the HPDEE technique can be employed as a promising approach for the targeted extraction of RG-I-enriched TBP.

#### 3.2.3. Comparison of Molecular Weights and Monosaccharide Units of TBP-W, TBP-D, and TBP-PD

Both the FT-IR and NMR spectra of TBP-W, TBP-D, and TBP-PD were investigated and compared to reveal the influences of various methods on their primary chemical structures. The FT-IR spectra of TBP-W, TBP-D, and TBP-PD are presented in Figure 3C. The results revealed that similarly typical adsorption bands (e.g., 3434.3, 2929.0, 1743.0, 1630.0, 1438.3, 1237.2, 1098.3, and 1022.0 cm^−1^) that corresponded to complex pectic polysaccharides were found in TBP-W, TBP-D, and TBP-PD, suggesting that their chemical groups were not affected by different extraction methods. The absorption bands around 1800–1600 cm^−1^ are due to the vibrations of carbonyl double bonds (C=O) [15,19], which can be utilized for the estimation of the degree of esterification of complex pectic polysaccharides. Generally, the adsorption bands at 1743.0 cm^−1^ and 1630.0 cm^−1^ indicate the vibrations of COO-R groups and COO- groups, respectively [19,20]. The esterification degrees of TBP-W, TBP-D, and TBP-PD were estimated to be 42.13%, 24.24%, and 21.65%, respectively, according to the ratio of adsorption peaks at 1743.0 cm^−1^ and 1630.0 cm^−1^. These findings reveal that the DES-based extraction techniques could reduce the DE values of pectic polysaccharides, probably due to the promoted RG-I proportion. In addition, the differences found in the FT-IR spectra observed in the 1200–1000 cm^−1^ region indicate differences in the content of GalA [20]. Compared to TBP-D and TBP-PD, the content of GalA was higher in TBP-W, resulting in intense adsorption peaks at 1098.3 cm^−1^ and 1022.0 cm^−1^.

**Figure 4:**
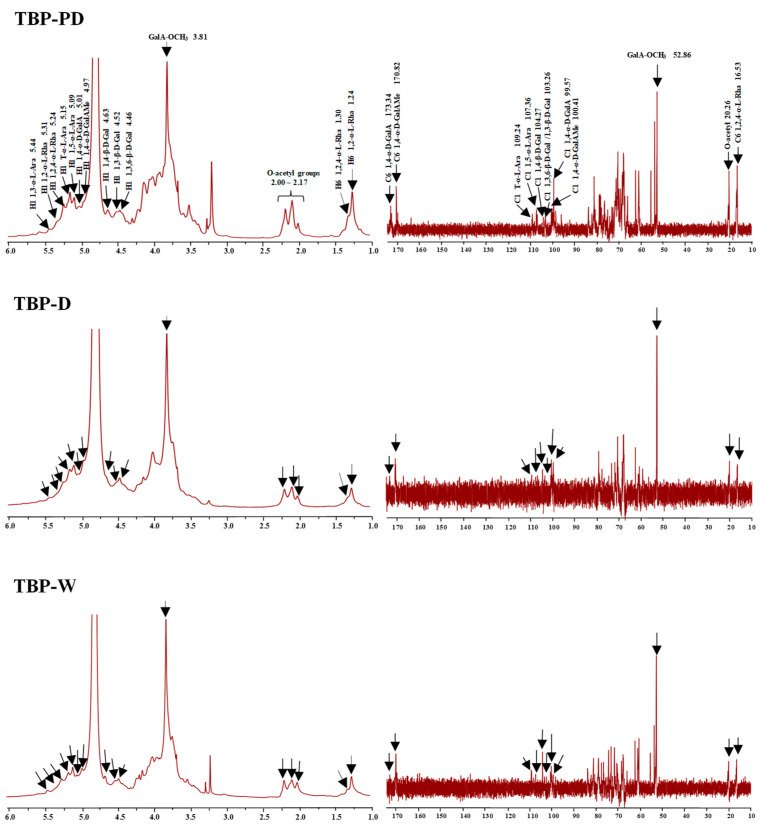
^1^H and ^13^C nuclear magnetic resonance spectra of TBP-W, TBP-D, and TBP-PD. The sample codes are the same as those in Figure 3.

To better understand the effect of various extraction methods on TBP’s chemical structures, 1D NMR analysis was performed. The ^1^H and ^13^C NMR spectra of TBP prepared using various methods are presented in Figure 4. The results show that their 1D NMR spectra were extremely similar, indicating that the different extraction methods did not significantly affect their primary chemical structures. Obviously, the typical 1D NMR signals of HG and RG-I domains were observed in all samples. For instance, the ^1^H signals ranging from 4.97 ppm to 5.44 ppm indicate α-D-GalA*p*, α-L-Rha*p*, and α-L-Ara*f* residues; the ^1^H signals ranging from 4.46 ppm to 4.63 ppm indicate β-D-Gal*p* residues; and the ^13^C signals ranging from 100.41 ppm to 109.24 ppm indicate α-D-GalA*p*, β-D-Gal*p*, and α-L-Ara*f* residues. The typical signals of GalA-OCH_3_ were observed at 3.81 ppm and 52.86 ppm, further confirming that TBP-W, TBP-D, and TBP-PD were esterified pectic polysaccharides [19,20,32]. Furthermore, several weak signals corresponding to O-acetyl groups were found in the range of 2.00–2.17 ppm, suggesting that these pectic polysaccharides were partially O-acetylated in positions 2 and 3 [1,32]. The typical signals of 1,4-α-D-GalAMe*p* were found at 4.97 ppm (H-1), 100.41 ppm (C-1), and 170.82 ppm (C-6), while the signals of 1,4-α-D-GalA*p* residue were found at 5.01 ppm (H-1), 99.57 ppm (C-1), and 173.34 ppm (C-6) [22,32]. The typical signals of 1,2-α-L-Rha*p* and 1,2,4-α-L-Rha*p* were detected at 5.31 ppm and 5.24 ppm (H-1) as well as 1.24 ppm and 1.30 ppm (H-6), respectively [32]. These findings reveal that both HG and RG-I regions existed in all samples. In addition, the typical signals of T-α-L-Ara*f*, 1,3-α-L-Ara*f*, and 1,5-α-L-Ara*f* were found at 5.15 (H-1)/109.24 ppm (C-1), 5.44 (H-1) ppm, and 5.09 (H-1)/107.36 (C-1) ppm, respectively [22,32]. The typical signals of 1,4-β-D-Gal*p*, 1,3-β-D-Gal*p*, and 1,3,6-β-D-Gal*p* were observed at 4.63 (H-1)/104.27 (C-1) ppm, 4.52 (H-1)/103.26 (C-1) ppm, and 4.46 (H-1)/103.26 (C-1) ppm, respectively [32,33,34,35]. These findings indicate that arabinan, type I arabinogalactan (AG-I), and type II arabinogalactan (AG-II) may exist as the branched chains of the RG-I pectic region. In conclusion, all the results show that HG and RG-I regions were observed to be the main pectic polysaccharides in TBP-W, TBP-D, and TBP-PD, and the proportion of RG-I was higher in TBP-PD than in TBP-W and TBP-D based on their monosaccharide ratios, chemical groups, and glycosidic linkages. Nevertheless, due to the limitation of 1D NMR analysis, further 2D NMR analysis is required to reveal more detailed chemical structures of TBP in future studies.

### 3.3. Comparison of Antioxidant Activities of TBP-W, TBP-D, and TBP-PD In Vitro

**Figure 5:**
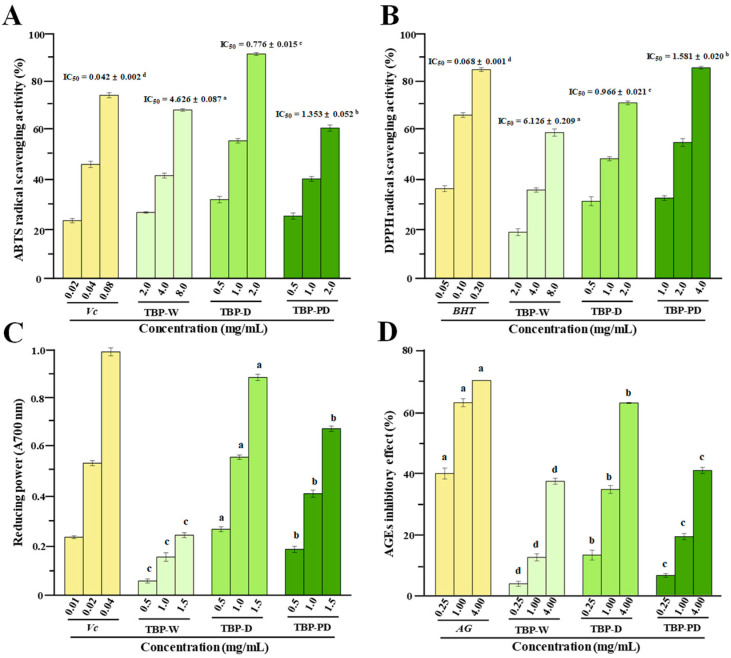
In vitro antioxidant (**A**–**C**) and anti-glycosylation (**D**) effects of TBP-W, TBP-D, and TBP-PD. The sample codes are the same as those in Figure 3. Vc, vitamin C. BHT, butylated hydroxytoluene. AG, Aminoguanidine. The error bars are standard deviations. Significant differences (*p* < 0.05) among different samples at the same concentration are shown by data bearing different letters (a–c). Significant differences (*p* < 0.05) among different samples and positive controls at the same concentration are shown by data bearing different letters (a–d).

Previous studies have shown that dietary polysaccharides derived from Tartary buckwheat seeds, sprouts, and green leaves can scavenge various free radicals, e.g., ABTS, DPPH, and nitric oxide (NO) radicals [9,36,37]. Therefore, the present research examines the antioxidant capacities of TBP-W, TBP-D, and TBP-PD. Figure 5A–C present the antioxidant capacities of TBP prepared using various extraction methods. The results show that all the samples had notable antioxidant properties when compared to the positive controls. Specifically, the IC_50_ values of the scavenging ability of TBP-W, TBP-D, and TBP-PD against ABTS radicals were 4.626 ± 0.087 mg/mL, 0.776 ± 0.015 mg/mL, and 1.353 ± 0.052 mg/mL, respectively. The IC_50_ values of the scavenging ability of TBP-W, TBP-D, and TBP-PD against DPPH radicals were 6.126 ± 0.209 mg/mL, 0.966 ± 0.021 mg/mL, and 1.581 ± 0.021 mg/mL, respectively. In addition, the FRAP values of TBP-D and TBP-PD at the concentrations of 0.5, 1.0, and 1.5 mg/mL were higher than those of TBP-W. Based on the above results, it is clear that the antioxidant activity of TBP-D and TBP-PD was significantly stronger than that of TBP-W, and the highest antioxidant activities among the three samples were observed in TBP-D. These findings indicate that the DES-based extraction approaches can extract pectic polysaccharides with superior antioxidant capacities [17,18]. In general, the antioxidant activities of pectic polysaccharides are frequently linked to their phenolic groups (e.g., bound phenolic acids and bound flavonoids), negative charges (e.g., free carboxyl groups and sulfate groups), and molecular weights [30]. Consequently, the highest antioxidant activities observed in TBP-D among the three samples could be partially associated with its higher free uronic acid and bound polyphenolics levels and lower molecular mass (Table 3).

### 3.4. Comparison of Anti-Glycosylation Activities of TBP-W, TBP-D, and TBP-PD In Vitro

Generally, advanced glycation end-products (AGEs) can be generated by complex aminocarbonyl reactions, and they can cause oxidative stress and the onset of a number of diseases [38]. Pectic polysaccharides can suppress the generation of AGEs during the Maillard reaction owing to their remarkable antioxidant properties [38]. Recent studies have unveiled that complex pectic polysaccharides from Tartary buckwheat sprouts and green leaves have potential anti-glycosylation effects [9,36]. Consequently, the in vitro anti-glycosylation activities of TBP prepared via various extraction methods were studied. Figure 5D presents the inhibitory effects of TBP-W, TBP-D, and TBP-PD against the generation of AGEs in the bovine serum albumin–glucose model, which revealed that all the samples exhibited potential anti-glycosylation effects. Indeed, the inhibitory effects of TBP-D against the generation of AGEs were greater than those of TBP-W and TBP-PD. More specifically, the IC_50_ values of TBP-W, TBP-D, and TBP-PD against the formation of AGEs were measured to be 8.086 ± 0.047 mg/mL, 2.355 ± 0.048 mg/mL, and 7.238 ± 0.135 mg/mL, respectively. Generally, pectic polysaccharides can eliminate free radicals during the generation of AGEs owing to their remarkable antioxidant effects [38]. Furthermore, it has been demonstrated that there is a positive correlation between the anti-glycosylation effect of pectic polysaccharides and their antioxidant activity [9,36]. Thus, the higher anti-glycosylation effect of TBP-D might be partially associated with its higher antioxidant capacity.

### 3.5. Comparison of Immunostimulatory Effects of TBP-W, TBP-D, and TBP-PD

**Figure 6:**
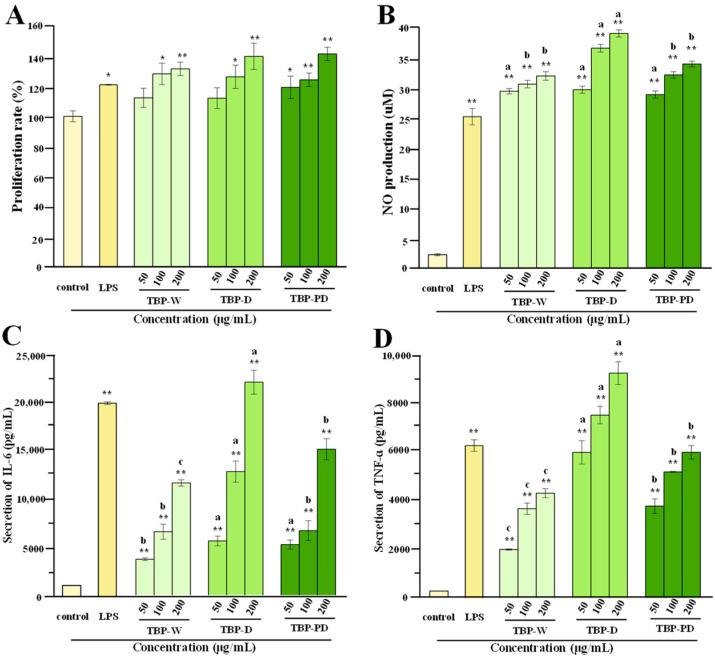
Immunostimulatory effects of TBP-W, TBP-D, and TBP-PD. The sample codes are the same as those in Figure 3. (**A**–**D**) indicate effects of TBP-W, TBP-D, and TBP-PD on the cell viability, NO production, secretion of IL-6, and secretion of TNF-ɑ of RAW 264.7 macrophages, respectively. The error bars are standard deviations. Significant differences (*p* < 0.05) among different samples at the same concentration are shown by data bearing different letters (a–c). Significant differences between the blank control group and the sample group are shown by * *p* < 0.05 and ** *p* < 0.01.

Generally, the immune system comprises body defenses against foreign or probably dangerous invaders. Pectic polysaccharides can maintain host health by activating the immune system, directly or indirectly interacting with it, thus activating several cellular events [39]. As shown in Figure 6A–D, TBP-W, TBP-D, and TBP-PD had no cytotoxic effects on RAW 264.7 cells, all of which exhibited notable enhancements in the production of NO, IL-6, and TNF-α in a dose-dependent manner. In detail, at a concentration of 200 µg/mL, the levels of NO, IL-6, and TNF-α released from RAW 264.7 macrophages induced by TBP-W, TBP-D, and TBP-PD were in the ranges of 30.35 ± 1.01–37.06 ± 1.29 µM, 12,464.83 ± 337.054–23,265 ± 1289.17 pg/mL, and 4269.55 ± 183.18–9173.18 ± 465.00 pg/mL, respectively. These findings indicated that the immunostimulatory effects of TBP-D and TBP-PD were stronger than those of TBP-W, suggesting that the optimized DESE and HPDEE techniques could be potential extraction methods for extracting TBP with superior immunostimulatory effects.

Generally, the immunostimulatory effects of pectic polysaccharides are linked to their structural properties [39]. As a consequence, the differences found in the immunostimulatory effects of TBP-W, TBP-D, and TBP-PD are due to their complex chemical structures. Previous reports have demonstrated that the immunostimulatory effects of pectic polysaccharides are closely associated with the proportions of HG and RG-I regions [2,3]. A decrease in the HG domain in pectic polysaccharides, resulting in an increase in the RG-I domain, can improve their immunostimulatory effects [40,41]. Therefore, compared to TBP-W, the stronger immunostimulatory effects observed in TBP-D and TBP-PD might be partially attributed to the increased proportion of the RG-I domain, suggesting that both the DESE and HPDEE techniques could partially intensify the immunostimulatory effect of TBP by increasing RG-I content. Furthermore, the immunostimulatory effect of pectic polysaccharides is also associated with their molecular weight and degree of esterification. Therefore, a relatively low molecular mass and a suitable esterification degree might also contribute to the higher immunostimulatory effects of TBP-D and TBP-PD [42,43,44]. Nevertheless, due to the indigestibility of pectic polysaccharides in the upper gastrointestinal tract, further studies involving animal models should be carried out to confirm that whether TBP exerts immunostimulatory effects through the modulation of gut microbiota.

## 4. Conclusions

In this study, two DES-based extraction techniques (DESE and HPDEE) were developed for extracting TBP. Compared to the CHWE method, the DESE method could obviously enhance the extraction yield, while the HPDEE method could notably reduce the extraction time, resulting in an increase in extraction efficiency. Indeed, both the DESE and HPDEE methods could selectively extract RG-I-enriched TBP, and the proportion of RG-I domain in both TBP-D and TBP-DP obviously increased. Furthermore, both the DESE and HPDEE methods could promote the antioxidant and anti-glycosylation effects of TBP by increasing its proportion of free uronic acids and content of bound polyphenolics and reducing its molecular weight. Moreover, both the DESE and HPDEE methods could also intensify the immunostimulatory effect of TBP by increasing its proportion of RG-I domain. Collectively, these findings suggest that the developed DES-based extraction methods, especially the HPDEE method, are promising techniques for the selective extraction of RG-I-enriched TBP.

## Figures and Tables

**Table 1:** The Box–Behnken designs with independent variables and observed yields for deep-eutectic-solvent-assisted extraction (DESE) and high-pressure-assisted deep eutectic solvent extraction (HPDEE).

Experiments	Levels of Extraction Parameters (DESE) ^a^				Yields (mg/g)	Levels of Extraction Parameters (HPDEE) ^b^				Yields (mg/g)
	X_11_	X_12_	X_13_	X_14_		X_21_	X_22_	X_23_	X_24_	
1	0 (180)	1 (70)	−1 (30)	0 (90)	48.6	1 (13)	0 (55)	0 (40)	−1 (90)	28.3
2	−1 (150)	0 (55)	0 (40)	−1 (80)	35.9	0 (10)	1 (70)	−1 (30)	0 (110)	17.0
3	1 (210)	0 (55)	0 (40)	1 (100)	66.1	0 (10)	0 (55)	0 (40)	0 (110)	38.8
4	0 (180)	−1 (40)	0 (40)	1 (100)	60.4	−1 (7)	0 (55)	1 (50)	0 (110)	28.8
5	1 (210)	−1 (40)	0 (40)	0 (90)	58.9	0 (10)	0 (55)	−1 (30)	1 (130)	23.9
6	1 (210)	1 (70)	0 (40)	0 (90)	53.9	1 (13)	0 (55)	1 (50)	0 (110)	30.1
7	0 (180)	0 (55)	0 (40)	0 (90)	74.5	0 (10)	0 (55)	0 (40)	0 (110)	39.4
8	0 (180)	0 (55)	1 (50)	−1 (80)	30.7	1 (13)	1 (70)	0 (40)	0 (110)	22.3
9	0 (180)	0 (55)	1 (50)	1 (100)	57.1	−1 (7)	−1 (40)	0 (40)	0 (110)	31.6
10	0 (180)	1 (70)	0 (40)	1 (100)	49.8	−1 (7)	0 (55)	0 (40)	1 (130)	29.8
11	0 (180)	0 (55)	0 (40)	0 (90)	73.8	0 (10)	−1 (40)	−1 (30)	0 (110)	26.3
12	−1 (150)	0 (55)	0 (40)	1 (100)	61.9	0 (10)	0 (55)	0 (40)	0 (110)	39.5
13	0 (180)	−1 (40)	0 (40)	−1 (80)	41.1	1 (13)	0 (55)	0 (40)	1 (130)	30.4
14	0 (180)	1 (70)	1 (50)	0 (90)	36.8	0 (10)	0 (55)	−1 (30)	−1 (90)	22.9
15	1 (210)	0 (55)	1 (50)	0 (90)	57.6	0 (10)	−1 (40)	1 (50)	0 (110)	30.9
16	−1 (150)	−1 (40)	0 (40)	0 (90)	56.8	1 (13)	0 (55)	−1 (30)	0 (110)	27.1
17	−1 (150)	0 (55)	−1 (30)	0 (90)	63.2	0 (10)	0 (55)	1 (50)	1 (130)	29.5
18	0 (180)	0 (55)	0 (40)	0 (90)	72.9	0 (10)	0 (55)	0 (40)	0 (110)	38.5
19	0 (180)	0 (55)	0 (40)	0 (90)	75.8	0 (10)	−1 (40)	0 (40)	1 (130)	32.7
20	1 (210)	0 (55)	−1 (30)	0 (90)	61.1	0 (10)	1 (70)	1 (50)	0 (110)	18.9
21	0 (180)	0 (55)	−1 (30)	1 (100)	64.5	−1 (7)	0 (55)	0 (40)	−1 (90)	24.6
22	0 (180)	0 (55)	0 (40)	0 (90)	72.3	1 (13)	−1 (40)	0 (40)	0 (110)	32.1
23	0 (180)	−1 (40)	−1 (30)	0 (90)	55.5	−1 (7)	1 (70)	0 (40)	0 (110)	19.9
24	0 (180)	−1 (40)	1 (50)	0 (90)	53.6	−1 (7)	0 (55)	−1 (30)	0 (110)	23.7
25	0 (180)	1 (70)	0 (40)	−1 (80)	24.3	0 (10)	1 (70)	0 (40)	1 (130)	19.3
26	0 (180)	0 (55)	−1 (30)	−1 (80)	39.7	0 (10)	0 (55)	0 (40)	0 (110)	39.2
27	−1 (150)	0 (55)	1 (50)	0 (90)	50.9	0 (10)	0 (55)	1 (50)	−1 (90)	25.1
28	−1 (150)	1 (70)	0 (40)	0 (90)	40.9	0 (10)	−1 (40)	0 (40)	−1 (90)	27.5
29	1 (210)	0 (55)	0 (40)	−1 (80)	43.3	0 (10)	1 (70)	0 (40)	−1 (90)	18.7

^a^ DESE: X_11_, extraction time (min); X_12_, water content in DES solvent (%, *v*/*v*); X_13_, liquid/solid ratio (mL/g); X_14_, extraction temperature (°C). ^b^ HPDEE: X_21_, extraction time (min); X_22_, water content in DES solvent (%, *v*/*v*); X_23_, liquid/solid ratio (mL/g); X_24_, extraction pressure (MPa).

**Table 2:** Analysis of the variance for the fitted second-order polynomial models for deep-eutectic-solvent-assisted extraction (DESE) and high-pressure-assisted deep eutectic solvent extraction (HPDEE).

	DESE				HPDEE					
	Sum of Squares	d*f*	Mean Square	*F* Value	*p* Value	Sum of Squares	d*f*	Mean Square	*F* Value	*p* Value
Model	52.4800	14	3.7500	142.9300	<0.0001	12.3700	14	0.8839	534.4000	<0.0001
X_11_ (X_21_)	0.8164	1	0.8164	31.1300	<0.0001	0.1180	1	0.1180	71.3500	<0.0001
X_12_ (X_22_)	4.3200	1	4.3200	164.7200	<0.0001	3.5200	1	3.5200	2128.7700	<0.0001
X_13_ (X_23_)	1.7600	1	1.7600	66.9400	<0.0001	0.4181	1	0.4181	252.8100	<0.0001
X_14_ (X_24_)	17.4700	1	17.4700	666.2000	<0.0001	0.2852	1	0.2852	172.4400	<0.0001
X_11_X_12_ (X_21_X_22_)	0.2970	1	0.2970	11.3300	0.0046	0.0090	1	0.0090	5.4600	0.0349
X_11_X_13_ (X_21_X_23_)	0.1936	1	0.1936	7.3800	0.0167	0.0110	1	0.0110	6.6700	0.0217
X_11_X_14_ (X_21_X_24_)	0.0256	1	0.0256	0.9761	0.3399	0.0240	1	0.0240	14.5300	0.0019
X_12_X_13_ (X_22_X_23_)	0.2450	1	0.2450	9.3400	0.0085	0.0182	1	0.0182	11.0200	0.0051
X_12_X_14_ (X_22_X_24_)	0.0961	1	0.0961	3.6600	0.0763	0.0529	1	0.0529	31.9800	<0.0001
X_13_X_14_ (X_23_X_24_)	0.0064	1	0.0064	0.2440	0.6290	0.0289	1	0.0289	17.4700	0.0009
X_11_^2^ (X_21_^2^)	2.4500	1	2.4500	93.3100	<0.0001	1.2200	1	1.2200	740.1300	<0.0001
X_12_^2^ (X_22_^2^)	14.3600	1	14.3600	547.6000	<0.0001	4.4500	1	4.4500	2689.8500	<0.0001
X_13_^2^ (X_23_^2^)	6.5400	1	6.5400	249.4300	<0.0001	3.5600	1	3.5600	2151.4900	<0.0001
X_14_^2^ (X_24_^2^)	15.8000	1	15.8000	602.2700	<0.0001	2.6100	1	2.6100	1578.4900	<0.0001
Residual	0.3672	14	0.0262			0.0232	14	0.0017		
Lack of fit	0.2919	10	0.0292	1.5500	0.3573	0.0161	10	0.0016	0.9082	0.5922
Pure error	0.0753	4	0.0188			0.0071	4	0.0018		
Correlation	52.8500	28				12.4000	28			

DESE: R^2^ = 0.9931, R^2^_adj_ = 0.9861, coefficient of variation (*CV*) = 2.97%, and adeq. precision = 43.0161; X_11_, extraction time (min); X_12_, water content in DES (%, *v*/*v*); X_13_, liquid-solid ratio (mL/g); X_14_, extraction temperature (°C). HPDEE: R^2^ = 0.9981, R^2^_adj_ = 0.9963, coefficient of variation (*CV*) = 1.44%, and adeq. precision = 76.2316; X_21_, extraction time (min); X_22_, water content in DES (%, *v*/*v*); X_23_, liquid-solid ratio (mL/g); X_24_, extraction pressure (MPa).

**Table 3:** Chemical compositions, molecular weights (*M_w_*), and constituent monosaccharides of pectic polysaccharides extracted from Tartary buckwheat green leaves using different methods.

	TBP-W	TBP-D	TBP-PD
Yields and chemical compositions			
Extraction yields (mg/g)	32.13 ± 0.55 ^c^	75.93 ± 0.91 ^a^	39.37 ± 1.03 ^b^
Total polysaccharides (mg/100 mg)	88.75 ± 0.95 ^b^	92.63 ± 3.01 ^ab^	94.48 ± 1.97 ^a^
Total uronic acids (mg/100 mg)	47.67 ± 0.74 ^a^	42.29 ± 0.41 ^b^	38.68 ± 1.41 ^c^
Total proteins (mg/100 mg)	1.95 ± 0.11 ^a^	1.49 ± 0.08 ^b^	1.88 ± 0.07 ^a^
TPC (mg GAE/g)	4.36 ± 0.21 ^c^	23.63 ± 0.69 ^a^	13.68 ± 0.25 ^b^
Degree of esterification (%)	42.13 ± 0.11 ^a^	24.24 ± 0.67 ^b^	21.65 ± 0.16 ^c^
Molecular weight and its distribution			
*M_w_* × 10^5^ (Da, error)	1.29 ± 0.01 ^a^	0.67 ± 0.01 ^c^	0.88 ± 0.01 ^b^
*M_w_*/*M_n_*	2.29	2.09	1.82
Monosaccharides and molar ratios			
Galacturonic acid (GalA)	3.35	2.34	1.71
Galactose (Gal)	1.83	1.61	1.56
Arabinose (Ara)	1.00	0.98	0.97
Rhamnose (Rha)	1.00	1.00	1.00
Xylose (Xyl)	0.14	0.16	0.17
Glucose (Glc)	0.22	0.19	0.24
Glucuronic acid (GlcA)	0.32	0.25	0.22
Mannose (Man)	0.32	0.31	0.31
MR1, Rha/GalA	0.30	0.43	0.58
MR2, (Gal + Ara)/Rha	2.83	2.59	2.53

TBP-W, TBP-D, and TBP-PD indicate pectic polysaccharides extracted from Tartary buckwheat green leaves using conventional hot water extraction, deep-eutectic-solvent-assisted extraction, and high-pressure-assisted deep eutectic solvent extraction, respectively. TPC indicates total polyphenolic content. mg GAE/g, mg of gallic acid equivalent per gram of polysaccharides. Superscripts (a–c) differ significantly (*p* < 0.05) among TBP-W, TBP-D, and TBP-PD.

## Data Availability

The original contributions presented in the study are included in the article, further inquiries can be directed to the corresponding author/s.
